# Twenty-Five Years of Endocrine Disruption Science: Remembering Theo Colborn

**DOI:** 10.1289/EHP746

**Published:** 2016-09-01

**Authors:** Carol F. Kwiatkowski, Ashley L. Bolden, Richard A. Liroff, Johanna R. Rochester, John G. Vandenbergh

**Affiliations:** 1 The Endocrine Disruption Exchange (TEDX), Paonia, Colorado, USA; 2 Department of Integrative Physiology, University of Colorado Boulder, Boulder, Colorado, USA; 3 Investor Environmental Health Network, Falls Church, Virginia, USA; 4 North Carolina State University, Raleigh, North Carolina, USA

## Abstract

For nearly 30 years, Dr. Theo Colborn (1927–2014) dedicated herself to studying the harmful effects of endocrine-disrupting chemicals on wildlife, humans, and the environment. More recently, she extended this effort to address the health impacts of unconventional oil and gas development. Colborn was a visionary leader who excelled at synthesizing scientific findings across disciplines. Using her unique insights and strong moral convictions, she changed the face of toxicological research, influenced chemical regulatory policy, and educated the public. In 2003, Colborn started a nonprofit organization—The Endocrine Disruption Exchange (TEDX). As we celebrate the 25th anniversary of endocrine disruption science, TEDX continues her legacy of analyzing the extensive body of environmental health research and developing unique educational resources to support public policy and education. Among other tools, TEDX currently uses the systematic review framework developed by the National Toxicology Program at the National Institute of Environmental Health Sciences, to answer research questions of pressing concern. In this article, we pay homage to the tenacious woman and the exemplary contribution she made to the field of environmental health. Recommendations for the future of the field are drawn from her wisdom.

## Introduction

The field of endocrine disruption is widely recognized as having formally begun in 1991 with the historic meeting at the Wingspread Conference Center in Racine, Wisconsin. This year, the National Institute of Environmental Health Sciences (NIEHS) celebrates the 25th anniversary of endocrine disruption science with an open meeting to explore past lessons and future directions (http://tools.niehs.nih.gov/conference/endocrine_2016/index.cfm). This anniversary provides an opportunity to reflect on the role of Theo Colborn, the woman who many referred to as “the mother of endocrine disruption.” In 1988, she saw the devastation of wildlife in the form of eggshell thinning, poor chick rearing, and the failure of offspring to thrive, and she feared for the health of humans. Prescient, tenacious, and passionate about the cause, she gathered the science and marched it to Washington, DC. When that was not enough, she rallied the masses with a hugely popular scientific detective story (Colborn et al. 1996). Although the development of the endocrine disruption movement was brought about by the contributions of many people (Schug et al. 2016), Theo Colborn was a seminal leader in the field (Grossman et al. 2015). In this article, we briefly chronicle her unique contribution, visionary leadership, and relentless voice for the health of future generations.

## Early Discoveries and Regulatory Response

The story of endocrine disruption began with research on the health of wildlife in and around the Great Lakes of North America, which had been accumulating industrial pollutants for decades (Canada-United States Collaboration for Great Lakes Water Quality 2012). Although the lakes had been intensely studied by world-renowned scientists, few understood the significance of the research as a whole. This changed in 1987 when Colborn joined a bi-national “State of the Great Lakes” environmental project (Colborn et al. 1990; Musil 2014).

Colborn brought to this task an intense curiosity and appetite for multidisciplinary learning. Only a few years earlier, in a striking career change at the age of 58, she earned a Ph.D. in zoology with minors in epidemiology, toxicology, and water chemistry. Before this career change, she was a pharmacist in New Jersey in the 1950s and then a sheep rancher in western Colorado, where she raised her four children. Her life’s wisdom combined with the enthusiasm of a new graduate gave her a unique perspective as she reviewed thousands of studies for the Great Lakes project. Not only did she voraciously consume the scientific literature, but she also networked with the many scientists whose studies she was reading—of which a handful had begun to sense the larger story. This rich cross-disciplinary conversation helped her connect patterns of health impacts that no scientist had previously made.

The result was what she would later label “that little grid that changed the world” (http://endocrinedisruption.org/about-tedx/theo-colborn). The grid featured 11 columns of health effects and 15 rows of wildlife species, with entries in the matrix denoting reported effects in each species. From bald eagles to beluga whales, mink to osprey, she showed the varying effects on reproduction, metabolism, and target organs. They were not all classic toxicological effects and were often only evident in the young. It became clear that chemicals were making their way up the food web, posing a threat not only to the health of the Great Lakes wildlife, but to people eating contaminated fish (Mehlman 1992).

To explore the research further, Colborn invited a small, carefully selected group of scientists to talk with each other face to face about their findings. In addition to wildlife biologists, she invited laboratory scientists, human health scientists, and others from 16 different disciplines, including endocrinology, toxicology, ecology, pharmacology, and anthropology. The meeting was held at the Wingspread Conference Center in Racine, Wisconsin, in July 1991. After several days of presenting their findings, the participants all agreed: “Many compounds introduced into the environment by human activity are capable of disrupting the endocrine system of animals, including fish, wildlife, and humans. The consequences of such disruption can be profound because of the crucial role hormones play in controlling development” (Mehlman 1992). It was at this meeting that the term “endocrine disruption” was coined. Perhaps more astonishing than such rapid cross-disciplinary consensus was that within five years of publication of what is now known as “The Wingspread Statement,” laws were being drafted to identify endocrine-disrupting chemicals (U.S. EPA 1998). Such was the power of the woman referred to as “the little old lady in tennis shoes” who was storming the halls of Congress in the early 1990s.

One of her demands was a shift in the emphasis in government chemical risk assessments from end points related solely to cancer to functional end points relevant to the endocrine system (Colborn 1995). This led to the creation, in 1996, of the Endocrine Disruptor Screening and Testing Advisory Committee, a group of multiple stakeholders tasked with advising the U.S. Environmental Protection Agency (U.S. EPA) on creating a new chemical risk evaluation process that would address the health outcomes resulting from exposure to endocrine disruptors.

Unfortunately, the scope of the tasks of the resulting Endocrine Disruptor Screening Program would prove to be impossible to execute because of the meager funds and short time frame allotted. In 2014, Colborn was still bemoaning the lack of appropriate government intervention to address what science had discovered to be numerous disorders associated with exposure to endocrine-disrupting chemicals.

## Educating the Public

Meanwhile, in 1996, Colborn and others were working to spread the word about endocrine disruption to a broader audience, beyond readers of scientific journals and academic texts. Colborn teamed with Dr. John Peterson Myers, director of the W. Alton Jones Foundation, and Dianne Dumanoski, an award-winning science journalist, to write *Our Stolen Future: Are We Threatening Our Fertility, Intelligence, and Survival? A Scientific Detective Story* (Colborn et al. 1996). In a forward by former Vice President Al Gore, he described it as a sequel to Rachel Carson’s *Silent Spring* (Carson 1962); the story drew worldwide attention and has since been translated into at least 14 languages.

After the publication of *Our Stolen Future,* Colborn dedicated the rest of her life to educating the public about endocrine disruption. In 2003, she moved back to western Colorado when health concerns forced her to retire from the pressures of working in Washington, DC. This move was far from a retirement: At the age of 76, Colborn started a nonprofit organization, naming it The Endocrine Disruption Exchange (TEDX). It seemed fitting that the first three letters of the acronym TEDX were the initials of her birth name.

During this time, the chemical bisphenol A (BPA) was rapidly emerging as a chemical of concern due to its estrogenic properties. Colborn immediately went to work reviewing hundreds of studies. She and her staff compiled the data on the adverse health impacts of exposure to low concentrations of BPA in laboratory animals (http://endocrinedisruption.org/endocrine-disruption/bisphenol-a/overview). They made the results publicly available in a spreadsheet that categorized the findings and presented summary statistics with very little interpretation so that readers could examine and utilize the data as needed.

Another visionary concept of Colborn’s that has become popular among scientists, academics, and students is referred to as “Critical Windows of Development” (http://endocrinedisruption.org/prenatal-origins-of-endocrine-disruption/critical-windows-of-development/overview). This interactive web page displays a timeline of the 38 weeks of human prenatal development. Superimposed on the timeline are studies indicating the disruptive effects of chemicals in laboratory animals. Thus, timing of normal developmental events in humans, such as the development of external genitalia, are chronologically aligned with the timing of chemical exposure in laboratory animals that leads to disruption, such as altered testes development. In addition to BPA, the effects of chlorpyrifos, dioxin, phthalates, and perfluorinated compounds are displayed on the timeline. A companion learning tool developed by TEDX is also available for college classroom exercises and discussion.

Colborn’s second major area of investigation arrived in the form of threats to the health of citizens in western Colorado from rapid expansion of hydraulic fracturing, a fossil fuel extraction process now commonly known as fracking. Again, her first response was to survey the literature and compile the data on the potential health impacts of chemicals used by the industry. At the time, there was little concern about the process or the use of chemicals in such close proximity to residences. Colborn’s commitment to public education drove her to travel the state of Colorado (then in her 80s) using an environmental justice grant from the U.S. Environmental Protection Agency (EPA) to give presentations to local communities about the process and potential harms from natural gas production in their neighborhoods. As with endocrine disruption, the issue quickly became political on a national scale.

In the eastern United States, exploration of the Marcellus Shale formation brought concerns for the safety of the water supply to New York City. TEDX was called upon to provide scientific information on the chemicals likely to be used and their potential health impacts. The New York City Water Board’s Impact Assessment, citing “potentially catastrophic consequences” reverberated throughout the state and led to an eventual moratorium on hydraulic fracturing in New York (New York Department of Environmental Protection and Hazen and Sawyer Environmental Engineers and Scientists 2009). Colborn also published two peer-reviewed papers on the subject: a review of the public health implications (Colborn et al. 2011) and a primary research investigation of air quality in a western Colorado community heavily impacted by oil and gas development (Colborn et al. 2014). These efforts had a tremendous impact on the field and helped mobilize community efforts to bring the issues to the attention of federal and state agencies.

## Continuing Colborn’s Legacy

On December 14, 2014, Theo Colborn died peacefully in her home at the age of 87. She worked steadfastly and passionately until the day she died. In an “In Memoriam” in the journal *Environmental Health Perspectives*, her significance to the field was lauded, with Linda S. Birnbaum, the director of NIEHS, among the co-authors (Grossman et al. 2015). TEDX provided an on-line forum for her many friends and colleagues to post comments on her influence on their lives. Eighty stories from around the world remain posted (http://endocrinedisruption.org/about-tedx/theo-colborn).

The synthesis of research continues to drive the efforts of TEDX to engage scientists, regulatory agencies, and the public. In 2013, moving beyond data compilation and narrative reviews, TEDX began using systematic review methods derived from a framework developed by the NIEHS, National Toxicology Program (NTP), Office of Health Assessment and Translation (OHAT). OHAT’s approach provides a thorough and transparent framework for identifying literature, evaluating risk of bias of research studies (i.e., internal validity), and combining data from epidemiological, *in vivo* animal models, and *in vitro* studies to determine whether a chemical poses a hazard to human health (NTP 2015). In addition, TEDX has advanced beyond the simple tools Colborn had at her disposal and is using custom systematic review software, including machine-learning, text-mining, and data extraction tools, to allow more rapid and comprehensive assessment of relevant literature. The organization is also conducting meta-analyses to provide quantitative estimates of effects.

Aspects of the systematic review were integrated into a TEDX review of the connections between BPA exposure and human health conditions (Rochester 2013). More than 90 peer-reviewed studies were included, attesting to the contribution of BPA to reproductive disorders, adverse birth outcomes, altered behavior in children, asthma, and metabolic disorders. The review also discussed the various mechanistic pathways that might support the biological plausibility of such findings in humans. In 2015, TEDX published a systematic review of the physiological impacts of two BPA analogs: bisphenol F (BPF) and bisphenol S (BPS). These chemicals are commonly used as substitutes for BPA, which is being phased out of some products due to the evidence of endocrine disruption. TEDX’s synthesis of the BPS and BPF studies have shown that these analogs are also endocrine disruptors that interact with the endocrine system via mechanisms similar to BPA (Rochester and Bolden 2015).

Systematic review methods were also used to assess potential endocrine disruptors for the International Chemical Secretariat’s Substitute It Now (SIN) List, which urges the business community to replace chemicals in their products that are likely to be regulated under Europe’s REACH legislation (http://chemsec.org/business-tool/sin-list/). The European Union is moving forward to adopt legally binding criteria to identify and regulate chemicals as endocrine disruptors. TEDX has contributed to such efforts by maintaining a publicly available list of potential endocrine disruptors (http://endocrinedisruption.org/endocrine-disruption/tedx-list-of-potential-endocrine-disruptors/overview) that has been used by many organizations, including the Joint Research Center of the European Commission and the NIEHS.

TEDX continues to address chemical exposure related to hydraulic fracturing. For example, it published a review of benzene, toluene, ethylbenzene, and xylenes (BTEX), which are products of oil and gas development. Exposure also comes from vehicular emissions and off-gassing of consumer products. The review concluded that BTEX may contribute to a variety of common health conditions including sperm abnormalities, reduced fetal growth, cardiovascular disease, respiratory dysfunction, asthma, and sensitization to common antigens (Bolden et al. 2015). Possible endocrine mechanisms were explored in the review. Notably, health effects of BTEX exposure were found at concentrations well below reference concentrations (i.e., safe levels) set by the U.S. EPA (Bolden et al. 2015). TEDX is also conducting a project to help prioritize chemicals that may be leading to endocrine, reproductive, birth, and long-term health effects in residents living near oil and gas development. This topic will be of continuing national concern as the Mancos Shale in western Colorado and eastern Utah was just determined to have the second largest reserve of technically recoverable gas after the Marcellus Shale formation in the eastern United States (Hawkins et al. 2016).

In addition to publishing in the peer-reviewed literature, TEDX also works to educate the general public. For example, TEDX organizes and hosts teleconference calls in which scientists present their findings and answer questions from the audience. These calls are recorded and available for listening at any time (http://endocrinedisruption.org/endocrine-disruption/videos-and-webinars). The calls address endocrine disruption and the health impacts associated with unconventional oil and gas development.

## Embracing the Future

As a testimony to how far the field has come in 25 years, it is remarkable to note that today several prestigious professional societies have called for preventive measures to avoid exposure to endocrine-disrupting chemicals. These include the Endocrine Society (Gore et al. 2015; Zoeller et al. 2012), the American College of Obstetricians and Gynecologists and the American Society for Reproductive Medicine (American College of Obstetricians and Gynecologists Committee on Health Care for Underserved Women et al. 2013), and the International Federation of Gynecology and Obstetrics (Di Renzo et al. 2015). Using their recommendations, TEDX is developing a project to provide practical advice to the general public on steps they can take to avoid exposure to endocrine-disrupting chemicals.

TEDX has recently evolved beyond the small town of Paonia in western Colorado, where it originated, and is currently developing a branch in North Carolina’s Research Triangle Park (RTP), a hub of environmental health research and the home of NIEHS and a major U.S. EPA research campus. RTP represents a unique blend of academia, government, and industry. Surrounded by three major research universities: North Carolina State University, the University of North Carolina, and Duke University, it is home to roughly 100 corporations and several government and nongovernment laboratories, including many related to toxicological and biomedical sciences.

The universities provide a steady stream of well-trained students to support the research laboratories in the area. On April 29, 2016, the NIEHS and the U.S. EPA co-hosted the 19th Annual NIEHS Biomedical Career Symposium at which TEDX participated in a panel on nonprofit organizations in the biosciences. TEDX plans to make use of the human, scientific, and technological resources as it puts down new roots in the RTP area.

## Conclusion

In one of her last public presentations, Colborn addressed President Barack Obama and the First Lady Michelle Obama of the United States in a direct, heartfelt, and poignant letter (https://www.youtube.com/watch?v=2r2Rx8VRq48). Identifying herself as a grandmother, she spoke of how little her generation worried about their children’s development beyond obvious birth defects. She expressed her dismay at how today’s parents must immediately fear the enormous odds of their children having attention deficit hyperactivity disorder, autism, asthma, diabetes, or obesity.

With her characteristic intensity and deeply knitted brow, she demanded a new program of “inner space” research, funded comparably to the country’s investment in exploring outer space. She envisioned a council of scientists, including endocrinologists, who understand the chemistry of the womb and how even small concentrations of endocrine-disrupting chemicals can cross the placenta and cause harm. Knowing she would not see such a council within her lifetime, she expressed the need for urgent action, more rapid than efforts to address climate change. Protection of the public health requires no less. Upon returning home she reflected that she had said her piece; it was up to the rest of us now.

Colborn’s letter to President Obama embodies everything that she stood for. It addresses policy at the very highest level, it demands inclusion of scientists in decision-making, and it is a personal appeal to the public to demand better protection from our government. Theo Colborn was an inspiration to all. She led a full life and then dedicated herself to public service, changing environmental health research as we know it. Motivated by her words, TEDX continues to inform and inspire scientific inquiry, deliver practical information to government decision makers, and expand public understanding of endocrine disruption, and does so all in an effort to reduce the risk posed by endocrine-disrupting chemicals to the health of humans and wildlife.
